# Single-Cell RNA Sequencing: Technological Progress and Biomedical Application in Cancer Research

**DOI:** 10.1007/s12033-023-00777-0

**Published:** 2023-06-15

**Authors:** Xu Chang, Yunxi Zheng, Kai Xu

**Affiliations:** Department of Otolaryngology, Head and Neck Surgery, The Second Affiliated Hospital of Nanchang University, Nanchang University, Nanchang, 330006 Jiangxi People’s Republic of China

**Keywords:** Single-cell RNA sequencing, Tumor heterogeneity, Microenvironment, Lineage tracing, Personalized medicine

## Abstract

Single-cell RNA-seq (scRNA-seq) is a revolutionary technology that allows for the genomic investigation of individual cells in a population, allowing for the discovery of unusual cells associated with cancer and metastasis. ScRNA-seq has been used to discover different types of cancers with poor prognosis and medication resistance such as lung cancer, breast cancer, ovarian cancer, and gastric cancer. Besides, scRNA-seq is a promising method that helps us comprehend the biological features and dynamics of cell development, as well as other disorders. This review gives a concise summary of current scRNA-seq technology. We also explain the main technological steps involved in implementing the technology. We highlight the present applications of scRNA-seq in cancer research, including tumor heterogeneity analysis in lung cancer, breast cancer, and ovarian cancer. In addition, this review elucidates potential applications of scRNA-seq in lineage tracing, personalized medicine, illness prediction, and disease diagnosis, which reveals that scRNA-seq facilitates these events by producing genetic variations on the single-cell level.

## Introduction

Cancer is a genetic illness that remains a major health risk for humans. As it develops, tumor cells acquire and accumulate oncogenic somatic mutations and non-genetic changes, which may promote unchecked cell growth, invasion, and resistance to treatment. The diagnosis, discovery of new drugs, design of clinical trials, and selection of therapy strategies all rely on an in-depth understanding of the molecular pathways driving carcinogenesis. Cancer genomes and transcriptomes, tumor development, intra-tumor heterogeneity, and treatment resistance are just a few of the areas where our knowledge of cancer biology has been bolstered by next-generation sequencing (NGS) in the last decade. Despite significant progress, most standard studies and datasets are based on bulk samples, which average the molecular features of cells in a tumor sample. The microenvironment of a tumor includes a wide variety of immunological, endothelial, and stromal cells, and is increasingly seen as an integral part of the tumor itself. Evidence is mounting suggesting cells in the tumor's microenvironment, in addition to cancer cells themselves, influence the tumor's biology and clinical behavior. This finding shows that bulk tumor profiling may hide cellular diversity, making it difficult to investigate the unique molecular pathways by which various tumor cell types may contribute to carcinogenesis. Therefore, many cancer researchers have used the newly available single-cell sequencing technologies to comprehend tumors at the level of individual cells to deal with the cell heterogeneity in tumors.

**Fig. 1:**
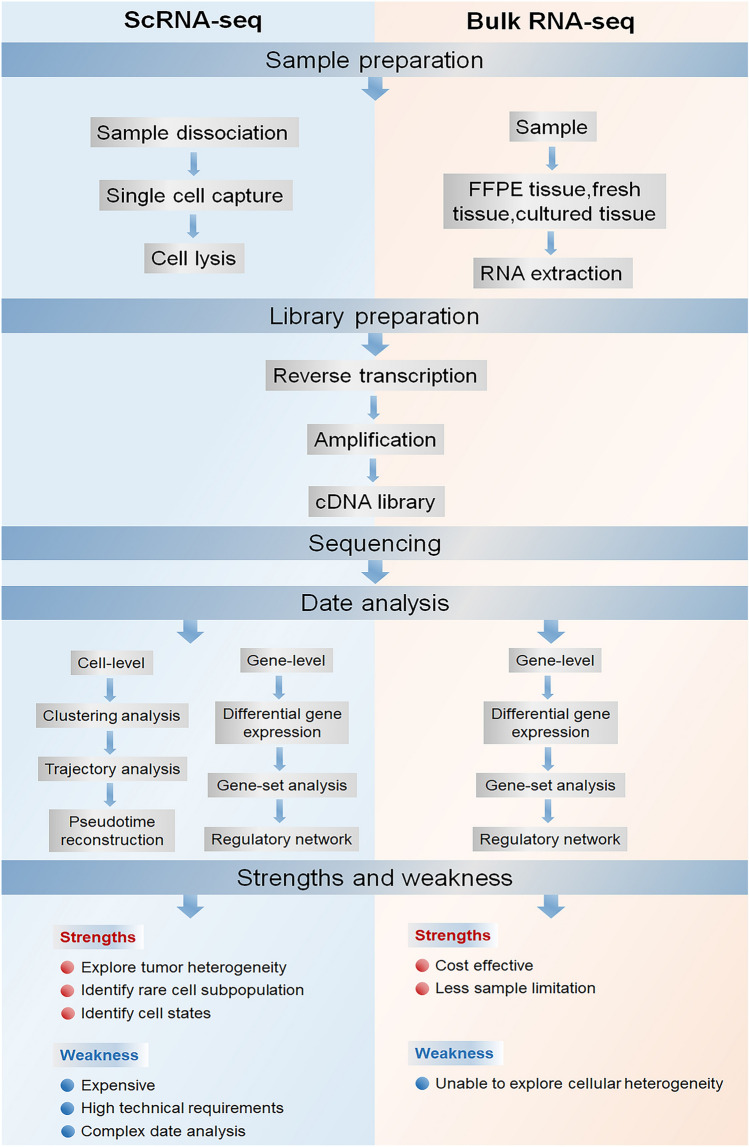
Comparison of working procedures of a typical single-cell RNA sequencing experiment and traditional bulk RNA analyses. The strengths and weaknesses of each method are also listed. *FFPE* formalin-fixed paraffin-embedded

The use of single-cell RNA sequencing (scRNA-seq) is a contemporary NGS approach that facilitates the identification of variations in genetic and protein expression among individual cells. This technology facilitates the acquisition of genetic data from microorganisms at the individual cellular level, thereby enhancing comprehension of the microenvironment. scRNA-seq has emerged as an influential innovation due to the advent of high-throughput sequencing techniques and the development of microfluidics, allowing researchers to obtain high-quality single-cell samples and expose the individuality of every single cell [65]. Traditionally, scientists have analyzed pooled populations of cells in tissues and organs because of the limitation of techniques to thoroughly analyze each cell’s uniqueness [75]. The comparison of bulk-RNA seq and scRNA-seq has been illuminated in Fig. 1.

Recent technical developments have enabled far better resolution gene expression analysis than previously achievable. The transcriptome at the single-cell level was characterized using scRNA-seq in 2009 [117]. Various techniques enhance our knowledge of the biological information in single cells, including analyzing the genomic DNA sequence, mRNA expression, protein expression, and cellular metabolites. Technological breakthroughs in cell isolation, high-throughput sequencing methods, and data analysis software have made the scRNA-seq application a reality. All these technological advancements indicate that scRNA-seq is becoming an increasingly powerful tool for evaluating developmental, normal, and pathological processes [78].

As a component of NGS, scRNA-seq has a role in exploring cell heterogeneity and uncovering novel genetic traits associated with clinical outcomes [101]. The profiled cellular programs in tumor samples have integrated genetic, epigenetic, and environmental cues, significantly improving our understanding of cancer heterogeneity [11], the cancer microenvironment [107], cancer metastasis [4], and cancer evolution in response to therapy [45]. Human hematopoietic development follows a similar pattern. For ethical reasons, it is difficult to acquire information on the lineage potential and hierarchical connections among early human hematopoietic progenitors [52, 56]. However, scRNA-seq can help to realize fate mapping and lineage tracing [127]. The molecular processes and sequence of events by which the ultimate identities of cells are determined during embryogenesis or remodeling may be inferred from the changes in cell states as tissues and organs develop [134]. For example, it may help to understand and control the fates of cells in vivo, anticipate where cancers and other diseases arise, and even recreate the differentiation process of cells [118]. scRNA-seq may be further utilized in inferring disease progression and prognosis in clinical trials [50], and single-cell genomics may reveal significant changes in the percentage and gene expression of cell types during injury and healing [21]. Furthermore, comparing the single-cell transcriptome between novel cell subsets under pathological states and normal cells facilitates the discovery of correlations between gene expression and phenotypes [87].

In general, the popularization of the scRNA-seq approach has been aided by the open sharing of methods, commercialization of technology, and widespread use in biomedical and clinical research. The primary single-cell sequencing technologies and applications, including tumor progression and lineage analysis, are outlined in this overview.

**Fig. 2:**
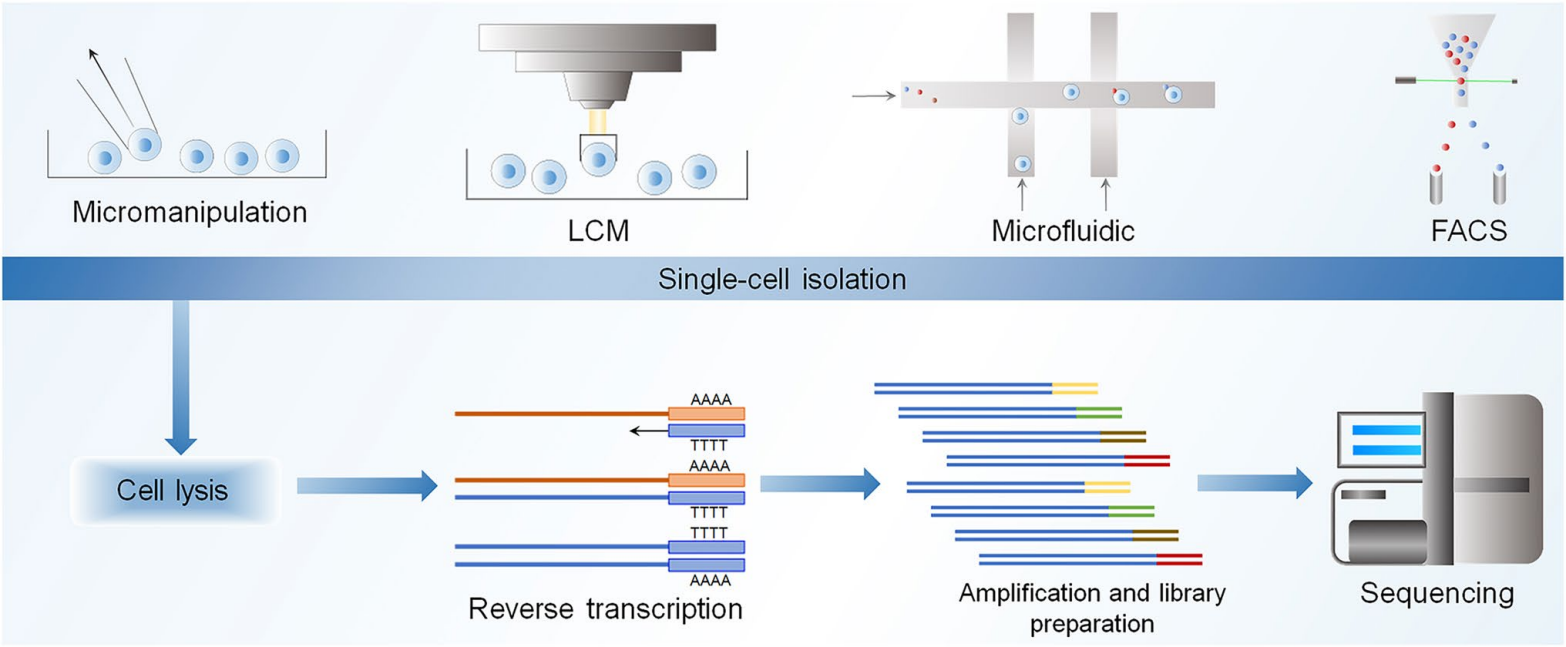
Pictures of single-cell RNA sequencing (scRNA-seq) experiments. A typical scRNA-seq workflow shares most of the following single-cell isolation by micromanipulation, laser-capture microdissection (LCM) microfluidics-activated cell sorting (FACS), (2) cell lysis, (3) reverse transcription of mRNA to cDNA, (4) cDNA amplification and library preparation, (5) sequencing

Conventional sequencing involves obscured signals from small cell populations. scRNA-seq is a set of techniques for untargeted measurement of novel genomic, transcriptomic, and proteomic information in individual cells [45]. In scRNA-seq, a single cell's DNA and RNA expression patterns can be determined by NGS, providing a better understanding of how a single cell functions and interacts with its environment. scRNA-seq technologies share common procedures, including isolating a single cell, RNA extraction, reverse transcription, preamplification, and data analysis [124]. The common workflow of scRNA-seq is illustrated in Fig. 2. Data analysis and the advent of cell separation have made it possible to create maps of individual cell types and states at the single-cell level [71].

The objective of our review is to provide a comprehensive overview of the advancements made in single-cell RNA sequencing (scRNA-seq) technology, encompassing the latest developments in emerging scRNA-seq methodologies. We conducted a comparative analysis of various scRNA-seq techniques to ascertain their respective strengths and limitations. Furthermore, our emphasis was primarily on the utilization of scRNA-seq in the identification of tumor heterogeneity and the characterization of the tumor microenvironment. Moreover, drawing from our independent investigation, our aim is to compile and condense the practical implementation of scRNA-seq in the context of tailored medical treatment, linkage mapping, and prognostication of illnesses.

## Single-Cell RNA Sequencing Technologies

### Isolation of Single Cells (Capturing Single Cells)

The entire procedure of single-cell RNA sequencing starts with the isolation of single cells from tissues. Single cells can be separated mechanically and enzymatically into different subpopulations without affecting their viability [101]. Micromanipulation is one of the traditional methods of single-cell isolation, and individual cells are isolated from microorganisms, solid histological tissue, or embryonic stem cells with a micropipette or forceps and microscope [48]. However, due to the difficulty of its operation, the labor involved, and the high potential for mechanical damage to targeted cells, this microscopic control system is rarely used [147].

Laser capture microdissection (LCM) focuses a laser beam on cells of interest and attaches them to membranes [38]. LCM is a powerful technique for isolating cell populations or regions of interest inside a tissue. The specified region is excised by a laser using direct viewing and can then be processed for a number of subsequent investigations. From DNA and RNA sequencing through mass spectrometry, this technology has been widely employed in the research of liver disorders. However, LCM has significant limitations. Due to the cost of the microdissection apparatus, this is an expensive technology. The yield and purity of DNA, RNA, and proteins extracted from improperly preserved and processed tissue samples may not be suitable for further analysis. The absence of a coverslip on the tissue slide may impede the accurate identification of the area to be microdissected. To preserve the integrity of the molecule of interest, optimum tissue preservation, precise tissue sectioning, and staining method optimization is necessary [99].

Initially labelingsing fluorescent monoclonal antibodies, the fluorescence-activated cell sorting (FACS) method primarily relies on flow cytometry. Negative selection may also be used to undetained populations [110]. Target cells are identified and separated using this method's analysis of their molecular markers and physical traits (such as cell size and fluorescence scattering) [55]. Nonetheless, FACS systems continue to be rather restricted in several applications. Because cells must be suspended, tissues must be dissociated, resulting in the loss of cellular activities, cell–cell connections, and tissue architecture [59]. Subpopulations with comparable marker expressions are difficult to distinguish, and the overlap of emission spectra amongst fluorochromes may enhance background noise, making low-intensity samples unidentifiable. Furthermore, as established by trypan blue exclusion and necrosis/apoptosis experiments for Chinese Hamster Ovary (CHO) cells and a human monocytic cell line (THP1), FACS sorting may have non-negligible impacts on cell survival [90].

The smallest sample volume for FACS systems is several hundred microliters to milliliters [55]. This is because lengthy tube sections often result in huge dead volumes, making it impossible to utilize uncommon samples, particularly when the full sample has to be evaluated [76]. Finally, because FACS systems generally have a complex system made up of non-disposable components, the sterile operation is challenging to achieve. However, such limits are not always present or significant, depending on the instrument, process parameters (such as speed, laser type, etc.), cell type, and application [47].

A microfluidic chip with microchannels that tightly control liquid flow can be employed during cell suspension preparation to achieve low sample consumption with microfluidic methods [97]. In the past few years, microfluidic procedures have gained popularity and are gradually replacing FACS and other previously used techniques for isolating cells.

Single-cell methods generally have their own unique advantages. The micromanipulator ensures the accurate collection of cells and minimizes cell damage. LCM collects spatial information and captures single cells from solid samples. FACS can focus on cells of interest. Microfluidic methods require a low volume of reagents, which makes them cost-effective. Understanding these single-cell isolation methods helps in choosing appropriate techniques for various cells from diverse targeted bulk tissues.

Following separation, identifying single-cell sequencing data is the most difficult aspect of this approach. The plate micro-reaction system and combinatorial index technology in single-cell barcode technology solve this problem and increase single-cell detection throughput by 100-fold. The plate-like micro-reaction system, for example, contains single cells, functional beads, and reverse transcription. Oligonucleotides such as primers, cell barcodes, and unique molecular identifiers (UMI), as well as 5' to 3' poly (dT), are used to modify the surface of functional beads. The UMIs target each molecule in a cell, unlike primers, polymeric (dT) fragments, and cellular barcodes, which are unique in each micro-reaction. A UMI can mark the DNA genome, transcriptome, immunological profile, and proteome [117].

Microsphere-based technologies like Drop-seq, Seq-Well, and inDrop are used to barcode specific molecules in single cells. After barcoding targeted molecules, a critical next step is the pre-amplification of transcripts by reverse transcription. Therefore, all RNA sequences are converted to cDNA for further amplification and sequencing. Finally, the sequencing results are integrated and analyzed by computers at different levels.

### Specific Protocols for Single-Cell RNA Sequencing

The first whole-genome amplification (WGA) technique developed was PCR-based amplification using Degenerate Oligonucleotide-Primed PCR (DOP-PCR) for single-cell analysis. This method is biased in amplification and PCR efficiency variations and has high error rates due to the thermostable polymerase and degenerate oligonucleotides used for primers [54].

The most widely used isothermal amplification method in WGA is multiple displacement amplification (MDA) [16]. The polymerase used is Φ29, with primary properties of excellent processivity and strand displacement activity. Since these enhancements improve genome coverage, there are fewer false-positive results. A proportionally more significant number of first-increased loci are overrepresented following exponential amplification,this overrepresentation is amplified further by greater fold amplification. Certain overrepresentation areas may have a systematic or stochastic bias [148]. Φ29 polymerase activity generates a tiny amount of chimeric sequence side products, which may be decreased by endonuclease treatment, allowing for the physical separation of amplicons through debranching [16].

PicoPLEX and multiple annealing and looping-based amplification cycles (MALBAC) integrate the advantages of the first two methods mentioned above. Both MDA and MALBAC can successfully amplify genomes from single cells. However, in both procedures, preventing the amplification of extraneous contaminating DNA is difficult when performed in microlitre reaction volumes in a tube. Additionally, a prior investigation discovered that MDA provides significantly greater genome coverage than MALBAC (72%) [54]. This causes sequence-dependent bias, which causes overamplification in some genomic regions and under-amplification in others. However, this sequence-dependent bias of MDA is not reproducible across the genome, making normalization impractical and CNV determination less precise. Despite this, MDA has been utilized extensively since its invention [148].

Overall, there are many factors to consider when deciding the best approach for a given clinical purpose. The technique used should be tailored to the application at hand, taking into account parameters such as the relative scarcity or abundance of the single cell to be separated, the ease with which DNA or RNA can be extracted from the target cell, and the needed depth of sequencing coverage. Although cost is always an essential concern with single-cell sequencing, it may be possible to obtain optimal results by considering all the above parameters. In Table 1, we summarize the most significant single-cell RNA sequencing methodologies and platforms. In Table 2, we enumerate and compare specific protocols for scRNA-seq to show their advantages and disadvantages.

**Table 1:** Collection of most important single-cell RNA sequencing techniques

Method	Technology	Name	Platform	Chemistry	References
scRNA-seq	Microdroplets	10× Genomics RNA	Commercial	3′ or 5′	Zheng et al. [148]
	Microdroplets	Drop-seq	Research	3′	Macosko et al. [86]
	Microdroplets	Indrop	Research	3′	Klein et al. [66]
	Nanowells	Seq-Well	Research	3′	Gierahn et al. [41]
	Nanowells	Takara Wafergen	Commercial	3′, full-length	Gierahn et al. [41]
	Nanowells	Cytoseq	Research	3′	Fan et al. [33]
	FACS	Smart-seq2	Research	Full-length	Ramsköld et al. [104]
	FACS	Sci-RNA-seq	Research	3′	Cao et al. [13]
scEpigenomics	Microdroplets	10× Genomics chromium ATAC	Commercial	Tagmentation	Satpathy et al. [108]
	FACS	ATAC tagmentation	Research	Tagmentation	Buenrostro et al. [10]
	FACS	dscATAC-seq	Research	Tagmentation	Cusanovich et al. [23]
	Microdroplets	scRBBS	Research	RBBS	Guo et al. [49]
scDNA-seq	Microdroplets	10× Genomics chromium CNV	Commercial	MDA	Andor et al. [2]
	Microdroplets	Mission Bio Tapestri	Commercial	Amplicon PCR	Lan et al. [74]
	Nanowells	Takara Wafergen	Commercial/research	Tagmentation	Laks et al. [72]
	Microdroplets	tagmentation	Research	Tagmentation	Zahn et al. [145]
	FACS	Sci-seq	Research	Tagmentation	Cusanovich et al. [23]

**Table 2:** Comparison of specific protocols for scRNA-seq

Kit	scRNA-seq method	Genome coverage (%)	Advantages	Disadvantages
Sigma-Aldrich	DOP-PCR [8]	39	Low genome coverage	Highly suited for evaluating CNVs with large bin sizes (1 million bases) on a broad genomic scale
Qiagen	MDA [82]	84	High genome coverage	Sequence-dependent bias, overamplification in certain genomic regions, and underamplification in other regions. Normalization becomes impossible, and CNV determination becomes less precise
Yikon	MALBAC [82]	72	CNV may be determined by doing signal normalization for noise reduction	Sequence-dependent bias, a high false positive rate for SNV detection, under-amplified regions of the genome are sometimes lost during amplification and cannot be accessed owing to the reproducible sequence-dependent bias

## Clinical Applications of Single-Cell RNA Sequencing

### Application of Single-Cell RNA Sequencing in Cancer Biology

**Fig. 3:**
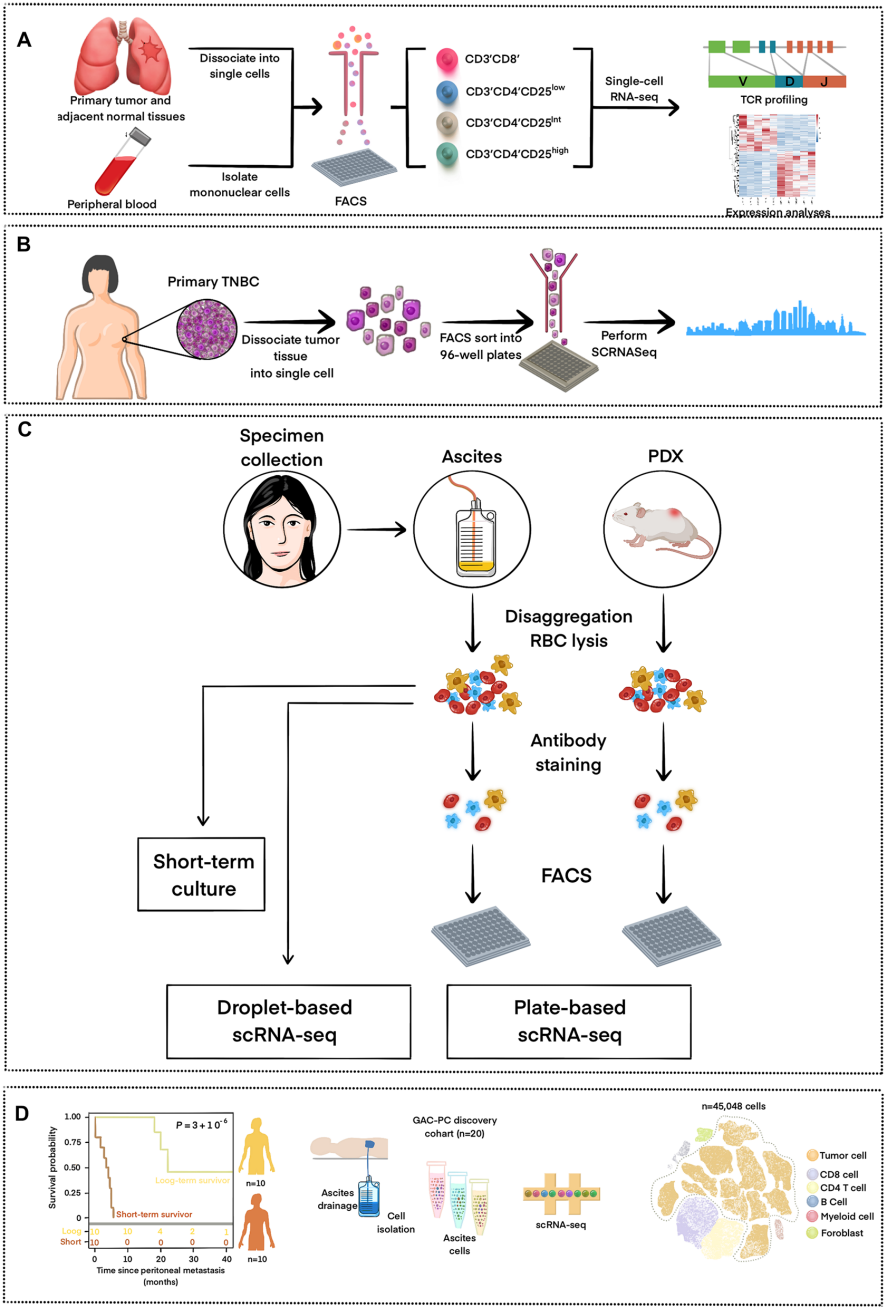
Summary of scRNA-seq applications in cancer research. **A** Non-small cell lung cancer. **B** Triple-negative breast cancer. **C** Ovarian cancer. **D** Gastric cancer

The development of scRNA-seq technology holds enormous promise for decoding cancer development, metastasis, and drug resistance. Based on genetic subtypes, scRNA-seq technology has been utilized to identify different cancer cell populations linked with poor prognosis and treatment resistance. By explaining tumor microenvironment heterogeneity, the approach has been utilized to identify unique immune cell subsets that may be implicated in tumor immunosurveillance and hence suggest prospective pharmaceutical targets [36]. In addition, a better understanding of the genetic composition of various malignancies has motivated clinical researchers to seek genotype-guided or individualized treatments [24]. Molecularly targeted medicines offer the best chance of treating cancer-related genetic abnormalities known as oncogenic drivers. The concept of targeted therapy emphasizes the correlation between the characterization of neoplasms and the unique therapeutic responses of individuals[103]. The study is grounded in the field of genomics and biomarker expression, indicating that genomic mutations and their consequent downstream pathways may serve as viable targets for pharmacological intervention or as indicators for prognostic purposes. The progress made in genome sequencing has facilitated the prompt identification of genetic disparities between cancerous and non-cancerous cells by researchers [53]. Several studies have shown that intra-tumoral heterogeneity causes cancer progression and enhances treatment resistance [24]. A detailed understanding of tumor dynamics is essential to designing effective and long-lasting treatments. Figure 3 illustrates the applications of scRNA-seq in cancer research.

#### Tumor Heterogeneity

The type of heterogeneity in a tumor population may be divided into intra-tumoral and inter-tumoral heterogeneity[26]. Many factors contribute to tumor heterogeneity across patients with the same kind of tumor, including genetic variations in the germline, shifts in the somatic mutational profile, and environmental influences [24]. When these characteristics are combined, they produce disparities between people, which is known as inter-tumoral heterogeneity. Intra-tumoral heterogeneity describes a patient's cancer cells [11]. The unequal distribution of genetically diverse tumour subpopulations across disease sites, as well as the dynamic changes in a tumor's genetic variety over time, are explained by cancer's spatial and temporal heterogeneity [75]. A few instances of how scRNA-seq may be used to interpret tumor heterogeneity are described below.

##### Non-small Cell Lung Cancer

Lung cancer is the leading cause of cancer-related fatalities in China, accounting for an estimated 61.02 million deaths [17]. Non-small cell lung cancer (NSCLC) represents 85% of lung cancer cases among the common subtypes. Improved knowledge of NSCLC pathogenic changes in genes, the discovery of novel drugs, and the use of biomarkers to identify individuals who are most likely to respond to immune checkpoint blockade therapy have all contributed to breakthroughs in NSCLC treatment [62]. For the vast majority of patients, cytotoxic chemotherapy remains an important component of systemic therapy, although immunotherapy and molecularly targeted medicines are now conventional first-line treatments for nearly half of those with advanced NSCLC[46].

Selective anaplastic lymphoma kinase (ALK) tyrosine kinase inhibitors (TKIs) have transformed ALK-rearranged non-small cell lung cancer therapy (NSCLC). ALK inhibitors include crizotinib, ceritinib, alectinib, brigatinib (2nd generation), lorlatinib, and ensartinib (3rd generation). Due to tumor cell heterogeneity, post-progression tumor tissues utilize diverse ALK inhibitor resistance pathways, and new diagnostic techniques are needed to understand resistance mechanisms and provide the best treatment.

Researchers wanted to know whether the sequencing of circulating tumor cells (CTCs) might provide further insight into ALK drug resistance variants and the possible tumor heterogeneity of ALK-rearranged NSCLC. After radiological disease progression on ALK-TKIs, blood samples from 17 patients with ALK rearrangement were used for CTC analysis. Multiple genetic variations in ALK-independent pathways were found by scRNA-seq in crizotinib-resistant CTCs [98]. The RTK-KRAS and TP53 pathways primarily showed mutations in EGFR, KRAS, BRAF, and other genes in CTCs, and the results were similar to those of ctDNA analyses. Nine variant genes in the RTK-KRAS pathway were detected in nine CTC samples. Among CTC mutations, EGFR, KRAS, and BRAF had mutation frequencies of 14.1%, 7.8%, and 6.2%, respectively. Both HIF and NOTCH pathways were altered in CTC-mutant samples, and both mutations were found in 5 of 9 individuals. These findings imply that the variability of the CTC genome in crizotinib-resistant ALK-rearranged cases is unexpected, including ALK-independent, RTK-KRAS, and TP53 pathways. Twenty genes were simultaneously altered, providing a crucial basis for treatment selection. These findings highlight the relevance of a single CTC gene in identifying drug-resistant cells in circulation.

The molecular features of the alveolar and cell damage repair signatures are noteworthy. Overexpression of SUSD2 and CAV1, WNT/β-catenin-associated pathway genes, was identified in an RD cohort. The WNT/β-catenin signaling pathway contributes to carcinogenesis, repair, and regeneration after cell injury in NSCLCs. AT2 cells, in particular, use the WNT/β-catenin signaling pathway for self-renewal and the damage response [91]. In addition, another research conducted by Yang et al. has shown that FOXP3 may increase the development of the β-catenin TCF4 complex and boost their role in activating EMT-related molecules such as snail and slug, resulting in the induction of EMT and the encouragement of NSCLC growth and metastasis[140]. Besides, Wang et al. revealed that SETD1A activates the Wnt/β-catenin pathway, which changes cancer stem cell characteristics as well as cisplatin sensitivity in NSCLC. SETD1A also interacts with and stabilizes β-catenin to activate the Wnt/β-catenin pathway through the SET domain. NEAT1 and EZH2 were revealed to be two novel SETD1A targets via which SETD1A enhances the Wnt/β-catenin pathway activity. In turn, β-catenin enhances SETD1A transcription, creating a positive feedback loop that promotes NSCLC development. Based on the results of this study, new prognostic markers and therapeutic targets may be found, potentially leading to advances in the diagnosis and prognosis of NSCLC patients [128].

In summary, the WNT/β-catenin pathway may be therapeutically targetable because it is involved in the transmission of signals for cellular harm and survival [70].

The EGFR-mutant NSCLC model and H3122 cells as an ALK fusion-driven NSCLC model have been used to assess the therapeutic potential of WNT pathway discoveries. The researchers expected that inhibiting WNT/β-catenin signaling and oncogenic EGFR or ALK would limit cell survival and enhance the depth of response from the start of therapy. Parental cells received an IC50 dosage of the relevant EGFR or ALK TKI (inhibitor concentration causing 50% cell loss) [70]. XAV939 and PRI-724, two WNT/β-catenin pathway inhibitors, were tested at four concentrations or in combination. In vitro, inhibiting the WNT/β-catenin pathway first followed by a TKI decreased cell confluency and increased response depth [70].

**Fig. 4:**
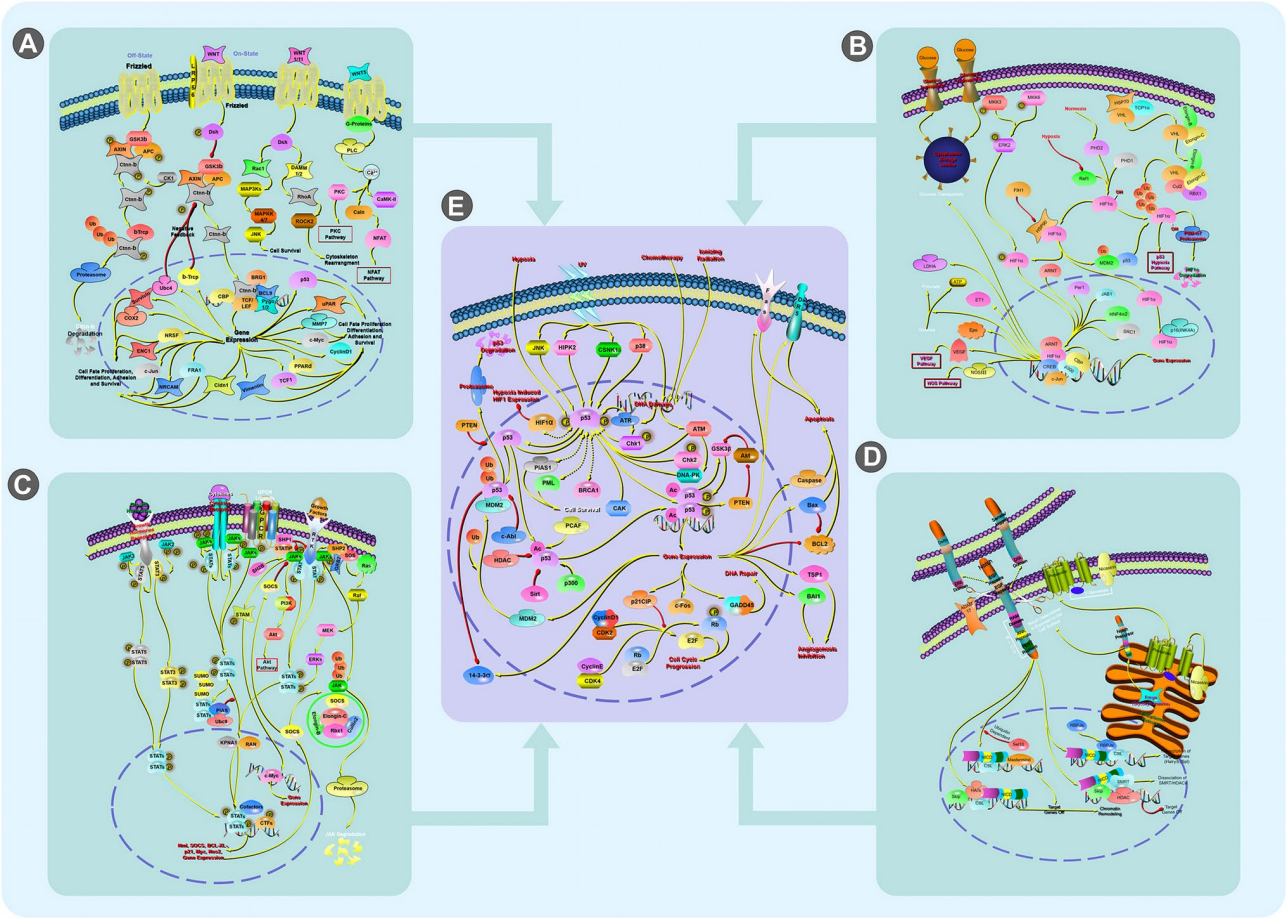
Collections of scRNA-seq applications in uncovering tumor heterogeneity in different kinds of cancers. Mainly altered signaling pathways related to different cancers are demonstrated in pictures. **A** WNT/β-catenin signaling pathway related to lung cancer. **B** HIF1-α signaling pathway associated with breast cancer. **C** Jak-STAT signaling pathway correlated with ovarian cancer. **D** Mainly changed Notch signaling pathway in gastric cancer. **E** The TP53 signaling pathway commonly alters in all these four kinds of cancers mentioned

By revealing the presence of not only the putative oncogenic driver but also additional oncogenic mutations, scRNA-seq data have demonstrated widespread intra-tumoral heterogeneity in oncogenic changes expressed in cancer cells. This might explain why a complete response to treatment is uncommon. Tumors contain the necessary genetic architecture and evolutionary playbook for resistance, and current bulk sampling studies may miss these “hard-wired” features. scRNA-seq profiling reveals the therapy-induced transcriptional plasticity that helps tumors to adapt and evolve. Transcriptional fingerprints for different treatment times and clinical situations were found. Most of these traits were biomarkers of lower OS, especially in PD. At RD, the alveolar cell signature was enhanced and associated with survival. This profile showed cellular plasticity and damage response, suggesting a treatment-induced adaptive phenotype that allows cancer cells to survive in a less aggressive state [130]. The alveolar cell signature and WNT/β-catenin pathway may also be involved in the damage response and regeneration. To improve cancer cell survival during therapy, the WNT/β-catenin pathway must be fine-tuned. Research suggests that manipulating cancer (or TME) cell fate(s) to exploit certain biological states might improve metastatic solid tumor treatment. Targeting cell state vulnerabilities or preventing adaptation may increase patient survival by preventing a tumor from acquiring total medication resistance [39, 89]. Authors have identified low T-cell infiltration in the TME of TN and PD patients, consistent with previous findings of little cytotoxic T-cell infiltration in treatment-naive EGFR mutant NSCLCs and a link between EGFR activation and immunosuppression in preclinical models. T-cell infiltration and lower immunosuppressive macrophage infiltration during RD on targeted treatment indicate an inflammatory phenotype. This inflammatory state may complement the cancer cell compartment's alveolar cell, damage repair, and regeneration states due to TME-cancer cell communication. Figure 4a shows the main altered lung cancer signaling pathways detected by scRNA-seq [27].

##### Triple-Negative Breast Cancer (TNBC)

High intra-tumoral variability is characteristic of triple-negative breast cancer (TNBC), a common subtype of breast cancer [143]. scRNA-seq analyses in TNBC have indicated subclonal heterogeneity and severe disease stages. Five distinct cell clusters were detected by scRNA-seq in untreated early TNBC tumors. Patients with TNBC have a high incidence of somatic mutations, frequent TP53 mutations (83%), and complex aneuploid rearrangements (80%), resulting in considerable intra-tumor heterogeneity [5].

On the basis of single-nucleus genome sequencing, the mutation rate in ER+ tumors is comparable to that in normal cells, and TNBCs exhibit sustained intra-tumoral variety [142]. According to marker genes and clustering, immune cells, and endothelial cells exhibit unique gene expression profiles. For instance, the majority of tumor epithelial cells exhibit luminal and luminal progenitor markers, though a minority express myoepithelial cell markers such as ACTA2 and TAGLN [19]. In addition, macrophages are more prevalent in CD45-unselected malignancies and CD45-selected tumors [1, 83]. In addition, the current research provides a genetic analysis of the luminal progenitors and hormone-sensing luminal cells, the two main subpopulations of the mammary luminal compartments. Overexpression of p53 and several activated p53 target genes is shown to be present in luminal progenitor cells, which are influenced by HGF/Met signaling in the mammary epithelium, as shown by the current research. Growing data indicate that p53 regulates stem/progenitor cell self-renewal, differentiation, and plasticity in both embryonic and adult tissues [60, 114]. In the absence of p53, luminal progenitors were amplified and their proliferative and self-renewal capacities were activated in vivo. Nonetheless, it had no effect on their inherent identity. Furthermore, luminal progenitors missing p53 and stimulated in vitro by HGF were unable to establish basal-specific features, demonstrating that p53 governs the adaptive nature of luminal progenitors after Met activation[20, 122]. Previous research has shown that the lack of p53 in the mammary epithelium facilitates the process of self-renewal and symmetric division of stem cells. The aforementioned process might explain why luminal progenitors lacking p53 have a restricted ability to undertake a luminal-to-basal transition after Met activation (64). Furthermore, another research found LGR5 activation in myoepithelial cells of regenerating mammary ducts[18]. In summary, luminal, luminal progenitor markers, and myoepithelial cell markers are significant in composing the genetic heterogeneity of the oncogenic pathway of breast cancer.

Intra-tumoral heterogeneity plays a critical role in the development of TNBC and contributes to the disease's resistance to treatment, metastasis, and poor prognosis. Indeed, a worse prognosis is expected for TNBC patients in whom even a fraction of breast cancer cells is not eradicated [5]. The whole single-cell exomes of 20 TNBC patients receiving neoadjuvant chemotherapy (NAC) were sequenced in a prior study. Genotypes associated with resistance to chemotherapy in TNBC were found to be both prevalent and adaptively selected by NAC [64]. CDH1 target profiles may provide a link between E-cadherin loss and the mesenchymal phenotype acquired by tumor cells in response to NAC [80]. In certain cases, resistance genes are present in a minute proportion of primed tumor cells.

Below, we highlight some important clinical implications of the scRNA-seq findings regarding tumor heterogeneity. Patients with TNBC can benefit from chemotherapy if the tumor mass contains certain genotypes, which can be diagnosed by detecting chemoresistant clones prior to initiating NAC treatment [84]. Such subgroups of TNBC may have prognostic relevance beyond histological approaches for determining clonal extinguishment or persistence as a result of genetic clones. Hence, it is important to consider all treatment options, including potential alternatives, before choosing one[31]. Chemoresistant phenotypes can be overcome, for instance, by employing therapeutic techniques such as targeting epithelial-mesenchymal transition (EMT) signaling or decreasing hypoxia with HIF-1 inhibitors [84]. Signaling pathways related to HIF-α are depicted in Fig. 4b.

Using scRNA-seq technology, a group of researchers developed a method for identifying and analyzing single cancer cells in the operation of micro-metastasis of breast cancer in human patient-derived xenograft (PDX) models[40]. These assays contributed to the comprehension of the change in gene expression that occurs during micrometastasis. Furthermore, researchers have identified mitochondrial oxidative phosphorylation (OXPHOS) as a crucial process in metastatic seeding [15]. In the preponderance of cases (e.g., breast tumors), this transcription factor does not promote clonal growth at the primary site, preventing positive selection. In contrast, RB1 loss and other oncogenic alterations that enhance OXPHOS (see previous subsection) promote both primary tumor growth and metastasis and therefore may be selected for during clonal evolution. This theory is further supported by the fact that slow-growing tumors, such as invasive lobular carcinoma (ILC), are highly metastatic. Moreover, inhibitors of the PI3K pathway inhibit tumor growth but reprogram mitochondrial trafficking, OXPHOS, and motility[81]. Contrary to the well-known stimulatory effect of hypoxia on tumor invasion, increased invasion as a result of elevated OXPHOS may manifest. However, tumor cells with elevated OXPHOS may be better able to adapt to hypoxia and migrate away from hypoxic regions [81]. In addition, another study has demonstrated the significance of OXPHOS in the development of malignancy. Tumor growth requires active mitochondrial function and oxidative phosphorylation (OXPHOS). Recent research indicates that the absence of the retinoblastoma (RB1) tumor suppressor increases mitochondrial protein translation (MPT) and OXPHOS in breast cancer [144]. Increased OXPHOS can increase anabolic metabolism and cell proliferation, as well as cancer stemness and metastasis. STAT3, FER/FER-T, and CHCHD2 are also involved in OXPHOS in mitochondria. Scientists postulate that RB1 loss is a prototypical oncogenic change that promotes OXPHOS, that aggressive tumors acquire lethal combinations of oncogenes and tumor suppressors that stimulate anabolism as opposed to OXPHOS, and that targeting both metabolic pathways would be therapeutic [69].

Oxidative phosphorylation may aid in metastasis [25]. OXPHOS-derived ATP can power cytoskeleton remodeling for motility or survival during anoikis during cell separation and migration. Since mitochondrial ROS-inducing mutations alone promote metastasis, OXPHOS-induced ROS generation may increase cell mobility by activating oncogenic signaling and promoting metastasis. In micrometastatic cells, NDUFS6, NDUFAB1, NDUFB2, NDUFAF4, UQCC3, and COA6, as well as the ATP synthase subunits ATP5I, ATP5G1, and ATP5J2, are upregulated. Micrometastatic cells additionally upregulate mitochondrial transport genes TOMM5, TOMM6, and TIMM13 and mitochondrial ribosome genes MRPL14, MRPL55, and MRPL51, which translate ETC protein-encoding mitochondrial genes. SOD1, which converts superoxide radicals to O2 and H2O2, is also present in higher levels in micro-metastatic cells. This enzyme may protect micro-metastatic cells from oxidative stress-induced death. Primary tumor cells contain more ALDOA, ALDOB, ALDOC, PGM1, and PGK1. Logistic regression research also indicates that LDHA is the top gene most descriptive of primary tumor cells because it increases aerobic glycolysis by speeding up the conversion of pyruvate to lactate and diverting it from the citric acid cycle (CAC). Cellular heterogeneity can impair medication efficacy. Scientists have discovered a unique kind of heterogeneity caused by temporary changes in glycolysis efficiency and ATP consumption or cycling in cells. Protein synthesis, glucose absorption, and the cell cycle combine to provide transitory resistance to acute metabolic challenges such as OXPHOS inhibitor therapy. In cancer and diabetes, OXPHOS suppression is beneficial, affecting metabolic stress signaling via AMPK, mTOR, and ERK. New AMPK activators and OXPHOS inhibitors may cure cancer, diabetes, and inflammatory illnesses.

##### Ovarian Cancer

Every year, 239,000 women worldwide are diagnosed with high-grade serous tubo-ovarian cancer (HGSTOC), which is characterized by a high recurrence rate and poor long-term survival [34]. scRNA-seq has recently enabled the investigation of the transcriptome diversity of tumors and associated stroma at a hitherto unexplored level in a variety of cancer types [9, 35]. The potential of this technique in ovarian cancer was recently established by Izar et al. [58], who described numerous cancer, fibroblast, and macrophage subpopulations using droplet-based scRNA-seq.

It is well known that fibroblasts promote tumor growth and metastasis. The epithelial-mesenchymal transition (EMT) and neo-angiogenesis are aided by cancer-associated fibroblasts (CAFs). In addition to CAFs, researchers have found that mesothelial cells and myofibroblasts, two additional fibroblast subclusters produced predominantly from unaffected tissue, are associated with poor outcomes. Mesothelium-derived fibroblasts were shown to co-express CALB2, WT1, MSLN, and keratins (KRT8, KRT18) [135]. All patient types and anatomical locations contained FB CALB2 cells, though normal and malignant omental tissue had the highest levels. Numerous pro-inflammatory cytokines (IL6 and IL18) and IL6-associated genes (COL8A1, CXCL16) were found in FB CALB2s (CCL2, CXCL1, IL6ST). IL6 promotes ovarian cancer cell proliferation, migration, neo-angiogenesis, and chemotherapy resistance, as shown by the fold increase in these factors [95]. Adipogenesis, bile acid, fatty acid metabolism, and cholesterol hemostasis were all shown to be activated during metabolic pathway analysis, along with the TNF-β pathway, the route responsible for IL6 synthesis. Using pySCENIC, researchers found transcription factors associated with adipocyte formation (SIX4, FOSL1) and fatty acid metabolism (NKX2-8) to be upregulated, along with the transcription factor STAT3, which is known to interact with IL6 [106].

As predicted, all three kinds of CAFs showed active EMT, as indicated by strong metalloprotease (MMP2, MMP14, MMP11) and collagen (COL10A1, COL11A1, COL5A1, COL1A1, COL27A1) production, allowing them to breakdown the extracellular matrix and leave the initial tumor location to metastasize. COL1A1, COL11A, and THBS1 are associated with tumor invasiveness and poor prognosis in ovarian cancer [133]. Despite gene expression similarities among CAF clusters, a metabolic pathway study showed that the TGF-β pathway drives EMT in CAFs. COMP, LTBP2, SKIL, TGFBI, PDGFC, and THBS1 were substantially expressed. CAFs also enhance glycolysis, hypoxia, and apoptosis. Cancer cell subclusters exhibited high EMT and IL2/STAT5 signaling, according to molecular pathway research. MMP2, MMP14, collagens, and conventional EMT markers (TWIS T1, ZEB1, WNT5A, and SNAI2) were increased, whereas epithelial markers were downregulated (absence of EPCAM). By sequencing numerous tumor samples, Langerhans-like dendritic cells and lipid-associated macrophages (MMP9) was found in the omentum. scRNA-seq of human omental adipose tissue has revealed that lipid-associated macrophages are the most numerous immune cell subgroup[61]. M_MMP9 macrophages express adipose tissue-specific genes (CD36, FABP4, FABP5) and TREM2, which is involved in phagocytosis, lipid breakdown, and pro-inflammatory mediator release [61]. Normal cells include many CAFs, including inflammatory CAFs that express high quantities of interleukin-6 and other cytokines that may cause cancer and treatment resistance. In addition, paracrine (and/or autocrine) signaling can synergistically stimulate the JAK/STAT pathway to cause malignant ascites and drug resistance in tumor cells and CAFs. The association of CAFs with peritoneal macrophages may also suppress or enhance tumor autonomic activities. IL-6-producing CAFs may help tumor cells to activate JAK/STAT, which is connected to poor prognosis and chemoresistance[29]. Blocking JAK/STAT boosts anti-tumour effectiveness in preclinical trials. Clinical trials such as phase I/II combination treatment should reveal ruxolitinib's role in HGSOC. CAF infiltration reduces the immune checkpoint inhibitor response rate 93. Overall, the tumour microenvironment affects numerous cancer treatments. Figure 4c shows ovarian cancer signaling pathway changes [12].

#### Gastric Cancer

Gastric cancer (GC) is the fifth most common illness and third leading cause of cancer death worldwide. Histopathology classifies GCs as intestinal or diffuse based on glandular cell morphology, differentiation, and cohesiveness. GCs are not isolated cancer epithelial cell masses. Instead, the tumor microenvironment (TME)—fibroblasts, endothelial cells, and immune cells—surrounds cancer cells in these tumors. Differential expression analysis has distinguished three epithelial cell types. The normal gastric epithelium has a pit, mucous neck, zymogen-secreting chief, intrinsic factor-producing parietal, and neuroendocrine cells. However, the GC type 1 and 2 subclasses show downregulated MUC6, TFF2, TFF1, and MUC5AC. The GC type 1 subclass overexpresses TFF3, FABP1, SPINK4, MUC13, and REG4. KRT7, KRT17, SOX4, and HES1, which are associated with metaplasia aetiology, are expressed at greater levels in the GC type 2 subclass. GC type 1 and type 2 cells overexpress gene sets for Myc, DNA repair, and Notch signaling. Only GC type 1 cells show enhanced EMT and KRAS signaling. Each patient's tumor includes many clusters of GC type 1 and 2 cells, showing sub-clonal heterogeneity. Pathway activation analyses divide these clusters into three to five subpopulations for each patient's tumor, indicating a subclonal nature. The cell cycle, KRAS pathway, and Wnt activity reveal P6207 heterogeneity. These subpopulations may help in tumor development. In one study, single-cell correlation analysis showed intra-tumoral heterogeneity in three patients. Bulk stemness, immunological, stromal, and tumor scoring showed a significant tumor and stromal score differences between primary tumor and metastatic tumor single cells, indicating compositional and functional changes in malignancies. A population-wide examination of TT and LN single cells found that primary cancer overexpresses NOTCH2, NOTCH2NL, KIF5B, and ERBB4 but that metastatic cancer overexpresses CDK12, ERBB2, and CLDN11 [6]. A heatmap of the top 50 highly expressed features from six redefined clusters using cell markers was created using tissue-specific markers,a heatmap of the top 100 highly expressed features based on clusters were produced from tissue-specific markers. Metastatic malignancies showed high ERBB2, CLDN11, and CDK12 levels, whereas primary tumors had high NOTCH2, NOTCH2NL, KIF5B, and ERBB4 levels. Gastric cancer cells express more Notch signaling pathway-associated proteins, such as Notch2, than normal tissues [146]. KIF5B and ERBB4 levels boost cancer cell proliferation. Cancer metastasis involves CDK12, ERBB2, and CLDN11. Studies have connected CLDN11 to tumor migration and metastasis [28]. Although the theorized "seed"-and-"soil" notion has yet to be fully described, stomach cancer aetiologies are partially known and empirically proven. It has been found that lymph node metastasis-prone subclones are more likely to possess CLDN11, a cell adhesion-related tight junction protein family member. Cancer cells invade lymph nodes from primary tissues. Future studies should examine transcriptomic, genomic, and geographical information from primary metastatic tumors to determine survival and evolution forces in the cancer-related microenvironment and uncover gastric cancer drivers [67, 138].

#### Tumor Microenvironment

Despite a major focus on malignant cells, non-cancerous cells, such as stromal and immune cells, play significant roles in tumor formation and treatment responses [126]. Ongoing evolutionary and ecological processes have been demonstrated, including constant interaction between cancer cells and the tumor microenvironment (TME). Non-cancerous host cells such as fibroblasts, endothelial cells, and neuroendocrine and adipocyte-derived immune system cells compose the TME. The extracellular matrix (ECM) and soluble products such as growth factors, hormones, cytokines, and extracellular vesicles are also present in the TME [73]. Understanding the molecular and cellular differences between tumor and normal host cells in the TME is essential. It is challenging to interpret processes and identify targets for intervention because of the wide variety of non-tumor host cells in the TME [137]. Therefore, deep sequencing at the single-cell scale is essential to deciphering the heterogeneity of the TME.

Based on a scRNA-seq analysis of human lung cancer, 40,250 different cells were collected to create a 52,698-cell TME transcriptome catalog [131]. Fifty-two stromal subgroups, including previously unknown fibroblasts, endothelial cells, and immune cells associated with tumors, were revealed. Compared with non-malignant counterparts and tumor tissue-associated counterparts, each subtype displayed distinct pathway activity [73]. Several stromal cell markers were negatively associated with patient survival regardless of tumor stage. Moreover, analysis of gene expression in the tumor stroma can identify therapeutic targets [88]. By using scRNA-seq, scientists have uncovered transcription factors that may transform anti-tumor cells into cancerous cells.

#### Single-Cell RNA Sequencing of Tumors and Personalized Therapy

scRNA-seq-based precision cancer treatment approaches can improve tumor diagnosis, prognosis, targeted therapy, early detection, and non-invasive monitoring [92]. With single-cell sequencing, rare variants, and specific cell expressions can be found with great accuracy. Rare tumor tissue variants can be identified, thereby advancing cancer genomics, drug resistance, and biomarker analysis [77]. More significant tumor heterogeneity is associated with poor prognosis, including treatment response, metastasis, and overall survival [92].

Founder gene mutations can be identified from the tumor phylogenetic tree to predict response to therapy. Analysis of individual cell sequences can uncover low-abundance variants that may help in identifying drivers of drug resistance. RNA-seq technology has successfully modeled drug resistance dynamics in breast cancer metastases [77]. After paclitaxel administration, cancer cells in metastatic disease stopped growing and died, but those resistant to the drug regrew. Researchers can elucidate genomic, epigenetic, and transcriptomic heterogeneity using the same cellular genome and transcriptome [139]. Drug development includes the following steps: discovery of drug targets, screening of drug candidates, determination of drug resistance, drug toxicity, and drug pharmacokinetics. Compared with massive genomic data, scRNA-seq for drug development can provide a deeper and more comprehensive understanding of responding and non-responsive individual cells. Therefore, scRNA-seq data are more efficient, accurate, and reliable than bulk genomic data. For example, scRNA-seq can identify drug candidates and targets resistance to drugs and toxicity.scRNA-seq technology enables early diagnosis and non-invasive monitoring of tumors. Finally, circulating tumor cells (CTCs) can provide insight into a metastatic spread [129]. The study of CTCs can also help to discover clones that invade surrounding tissues early in the tumor. Single-cell transcriptome data can reconstruct transcriptional dynamics during development, differentiation, and clonal evolution using algorithms such as Wanderlust or Monocle. It is possible to identify signature transcriptions of tumor states, which will significantly impact treatment decisions [132].

#### Tumor Cells and Immunotherapy Response

scRNA-seq has led to a unique insight into the tumor-internal and external pathways that determine response to and, ultimately, resistance to immunotherapy [57]. Through meticulous study of individual cells, genomics, transcriptomics, epigenomics, and proteomics provide a wealth of information for studying tumor molecular and cytology. As a result, single-cell immune and stromal cell assays are effective in identifying cell types and conditions associated with patients' shared immunotherapy responses. Research of the transcriptome of individual tumor cells allows for the classification of tumor types and cellular hierarchies [45], as well as the identification of transcriptome characteristics associated with treatment response or drug resistance.

For example, 31 melanoma patients were analyzed using scRNA-seq based on immune checkpoint inhibitor (ICI) therapy[45, 104]. The researchers discovered a transcriptional process in tumor cells that are involved in the resistance of T lymphocytes to therapy. Expression of CDK4 genes was enhanced, but that of genes encoding proteins such as interferon signal transduction or compliments was decreased. Additionally, a separate cohort of 112 patients showed reduced efficacy for PD-1 targeting. These findings demonstrate the value of small-scale single-cell investigations in deconvoluting large-scale data and in identifying therapeutically relevant cellular subpopulations [68, 88].

### Lineage Tracing

Lineage tracing attempts to construct a hierarchical tree of all descendants derived from a single cell, though it does not always incorporate positional information. By tracking the state of cells, the ultimate identification of cells in embryogenesis and regeneration can be understood [37]. In addition, this technology can be used for regulating cell fate, predicting tumor origin, and remodeling cell differentiation in vitro. In recent years, the development of high-throughput scRNA-seq technology has provided a complete transcriptional map of adult tissues and embryos based on millions of individual cells [127].

Currently, single-cell genomics methods allow for an objective diagnosis of cell identity by collecting thousands of gene expression measures while maintaining the cellular resolution required for proper lineage remodeling [63]. In addition to RNA capture, genomic technologies are currently used to measure the transcriptome, epigenome, and proteome. Advances in computer technology have enabled these data to be visualized and interpreted, resulting in new insight into biological processes and the identification of new cell types. Especially relevant to developmental biologists, single-cell data can infer the differentiation routes of cells [63]. Consequently, these approaches can help in identifying transcription factors associated with particular branches of differentiation.

In recent years, a large number of analyses of the single-cell transcriptomes of developing tissues and organs, including the inner ear, have suggested that scRNA-seq may be used to identify slight transcriptome differences between various cell types [30]. At E16.5-E18.5 d of mouse embryo development, epithelial cells isolated from the airways begin to differentiate towards the distal airway apex. In addition, they mainly differentiate into type I alveolar epithelial cells (AT1), which have a role in gas exchange, and type II alveolar epithelial cells (AT2), which produce surfactants. Quake et al. analyzed 80 single cells in the lung tissue of an E18.5 d mouse and found five cell clusters, four of which were composed of AT1, AT2, ciliated epithelium, and Clara cells. The fifth subgroup not only expressed AT1 and AT2 marker genes but also showed that progenitor cells with the characteristics of AT1 and AT2 may exist [123], which indicated that single-cell-resolution transcriptome sequencing may effectively promote the study of developmental biology [85]. By using the single-cell-binding RNA-seq method, the mechanism of mouse organogenesis has also been studied at single-cell resolution, providing a new holistic view of animal development. The above findings may also constitute a critical step towards understanding pleiotropic developmental disorders at the biological level and allow for a detailed study of the subtle effects of genes and regulatory sequences during development [14].

Owing to genome-scale single-cell analysis, our understanding has shifted over the past few years from an animation of discontinuous changes to a dynamic state driven by data [63]. These features not only predict the differentiation dynamics of thousands of genes but also infer new cell transitions and end states, interactions with the cell cycle, and the ability to "cycle between cell states." To draw an authentic lineage, the blood relationship of a cell needs to be understood. Therefore, future fate maps can provide powerful tools for tracing and reconstructing lineage relationships [125].

### Single-Cell RNA Sequencing and Disease Prediction

scRNA-seq can be used to identify novel cell types, analyze stem cell differentiation and single-cell trajectory construction, and compare healthy and disease-related tissues at a single-cell level. Recent advances in cardiovascular research demonstrate the importance of these applications, as evidenced by the generation of cell atlases for mammalian cardiovascular development and stem cell differentiation. To develop effective patient-specific therapeutic strategies, single-cell omics can be used in cardiovascular precision medicine to characterize responses of individual cells to drugs or environmental stimuli. Single-cell transcriptomics has also revealed cell subpopulations in adult mouse hearts. scRNA-seq of non-cardiomyocyte cells from undamaged adult mouse hearts showed an extensive intercellular communication network involving endothelial cells, cardiac fibroblasts, and immune cells.

Recent developments in scRNA-seq enable cell types and their significance in health and illness to be separated from complex tissues and host compartments[51]. This approach has altered our capability to understand the immune system in unprecedented detail, particularly with regards to processes like hematopoiesis, carcinogenesis, the lymph node compartment, and responses to microbial ligands [42, 102]. The ever-growing amount of data has given rise to analytical techniques like computational deconvolution procedures that forecast the precise compositions of cell types from large-scale gene expression data. These algorithms, however, are reliant on preexisting knowledge or particular datasets that are part of the experimental systems[100, 109].

#### Cardiac Homeostasis and Disease

scRNA-seq analysis of adult mouse hearts indicated a substantial intercellular communication network. Analysis of adult myocardium at homeostasis and after ischaemic injury using SORT sequences has revealed various known cell types. Additionally, Gladkar et al. suggested that ischemia–reperfusion induces cell subsets of many different cell types and that Ckap4 is elevated in activated fibroblasts [44]. To identify gene modules of hypertrophic cardiomyocytes, the Smart-seq2 platform has been employed to conduct scRNA-seq of adult cardiomyocytes at the single-cell level [96]. Moreover, droplet-based scRNA-seq was performed on endothelial cells to explore the endothelial heterogeneity of neovascularization after myocardial infarction [105], and plasmalemma vesicle-associated protein (Plvap) was identified as a novel marker of cardiac neovascularization. The combined data show that the intact single-cell properties of cardiac ECs can help to block new angiogenesis within the myocardium. This property may help to identify new therapeutic targets for heart diseases [105].scRNA-seq has also been used to evaluate gene expression in cardiomyocytes isolated from human embryos [22]. The developmental pathway of the human heart has been mapped using single-cell tagged reverse transcription sequencing with approximately 4,000 cardiac cells from 18 human embryos. Gene expression in both cardiomyocytes and fibroblasts was found to be gradually altered throughout development. Using human and mouse scRNA-seq data, cardiomyocytes, endothelial cells, fibroblasts, and epicardial cells were compared and showed the highest transcriptional similarity in cells from human and mouse cardiomyocytes [22]. The results demonstrate higher expression of THY1 in human fibroblasts,expression of CFB and ITLN1 was lower in mouse cardiomyocytes. However, Icam2 was only expressed in the endothelial cells of mice, whereas Rnf213 was expressed exclusively in the mouse epicardium, showing different gene expression patterns. Extensive work is underway to develop a single-scale atlas of the adult heart [112]. In general, scRNA-seq plays a vital role in understanding cellular variation and in identifying critical transcriptional processes associated with cardiac development and disease.

#### Coronary Vessel and Disease

The formation of coronary arteries is a dynamic and regular development process, and scRNA-seq technology plays a crucial role in explaining these dynamic cellular transformations. For example, combining scRNA-seq with genetically tracked genes in mice revealed that coronary arteries derive from the pre-artery, as they develop through a specialized population of veins [116]. scRNA-seq has also been used to characterize the cellular status and fate in significant vascular lesions. Cells such as vascular endothelial cells, vascular smooth muscle cells (VSMCs), and immune cells all display different plasticity and sensitivity to extracellular signals [79, 113]. Although VSMCs are terminally differentiated cells, they are highly plastic, and the use of single-cell analysis helps to delimit their ability to differentiate into fibroblast-like cell types. Additionally, single-cell analysis can help in determining the differentiation status of VSMCs in atherosclerosis [141].

## Challenges and Future Perspectives

The goal of scRNA-seq is to bring genomic studies to the cellular level, providing a new perspective on our understanding of genetics. These tools open up a new field of research to analyze the role of individual cells in ecosystems and biological biology. In recent years, there has been significant progress in obtaining high-quality single-cell data, enabling the discovery of new biological phenomena that conventional bulk genome queries cannot detect. Figure 1 illustrates the difference between traditional bulk RNA sequencing and single-cell RNA sequencing. The latter is a powerful tool to explore the complexity of cancer and its tumor environment to lead to personalized therapy. It also offers novel perspectives to investigate more ways of diagnosing and treating common diseases. In addition, scRNA-seq has been used to track cell development and stem cell research. However, this technology still faces several challenges.

Cell integrity and viability are critical for subsequent single-cell analysis, requiring that individual cells be separated quickly and accurately, with minimal damage to the cells. Therefore, to obtain high-quality scRNA-seq data, there are four major technical problems that need to be overcome: physical isolation of single cells; gene amplification of a single cell to obtain sufficient substances for further analysis; economical and efficient genome analysis queries to identify variants that validate research hypotheses; and analysis of introduced errors and biases. To maximize the quality of single-cell data and ensure that the signal is not affected by technical noise, each variable must be carefully considered when conducting single-cell experiments. The high cost of scRNA-seq is also a non-negligible problem. Although existing detection systems have brought the cost of sequencing each cell type to an acceptable level, the overall cost is still prohibitive because thousands of cells may need to be analyzed. Reducing the cost of sequencing will drive the implementation of scRNA-seq in oncology and other fields. Temporal and spatial measurement of the molecular profile by using in situ sequencing and real-time sequencing, as well as in vivo analysis of the DNA and RNA from single cells, have been developed, but these methods need enhanced sensitivity, coverage, accessibility, and cost reduction [93]. In addition, in short-read RNA-seq technology, errors and biases are mainly generated during the preparation of sequence libraries and assembly of short reads. These methods have difficulty in accurately identifying multiple different subtypes of a specific gene. Read coverage and sequencing depth must also be increased to overcome insufficient read length. Long-read RNA-seq technology overcomes the shortcomings of template expansion, reduces the false-positive rate, and can identify longer non-annotated transcripts, thus addressing the limitations of traditional short-read methods [32, 111]. However, this method is associated with problems of reduced throughput, high cost, and high sequence error, especially insertion and deletion. To reduce random errors, PacBio circular consensus sequencing (CCS) was developed, which can repeatedly read out a molecule over multiple cycles, increasing the depth of the sequence. However, this method also reduces the identification of specific isomers. In addition, the sensitivity of long-read sequencing to identify differentially expressed genes is lower than that of short-read sequencing [119]. Accordingly, a more complete and precise analysis can be obtained by hybridization of long-read and short-read sequencing [115].

The integration of artificial intelligence (AI) is increasingly being recognized as a pivotal resource in life science and healthcare research. Despite being a nascent field, research on artificial intelligence is transforming our comprehension and outlook on the scientific domain. According to recent estimates provided by the European Commission, AI-based medical startups receive approximately 13% of global venture capital investments, which amounts to €5 billion [7]. This dedication demonstrates the interest in the potential of artificial intelligence to improve healthcare. Precision medicine is a cutting-edge approach to treating disease. The generation of genomic Big Data (i.e., Big Data derived from genome sequencing), the gathering of clinical data, and the development of bioinformatics over the past ten years have made it possible to pinpoint the genetic factors underlying the onset and progression of diseases and to support clinical patient management. Personalised therapeutic treatments are still scarce despite the high expectations [43]. The inadequacy of AI infrastructure and models to support the ongoing production of large-scale genomic data represents a significant failure. Hence, the challenge lies in comprehending the heterogeneous information encompassed within this data [94, 121].

The high-throughput profiling of all RNA species generated by cells is known as transcriptomics. Transcriptomics has shown rapid growth in recent years among genetic Big Data [136]. RNA sequencing, also known as RNA-seq, is a technique that enables the characterization of dynamic biological processes that are currently active in a population of cells or in individual cells. The evaluation of the intricacy of these profiles has the potential to facilitate the identification of novel biomarkers and therapeutic targets. RNA-seq screenings are increasingly being integrated into precision medicine trials [120], AI mining of these data is thus required to determine novel clinical targets.

The increasing demand for artificial intelligence (AI) in the field of precision oncology will necessitate the presence of medical professionals and specialists who possess the ability to effectively interpret outcomes and make informed decisions regarding precision therapeutic interventions. Additionally, these individuals will play an active role in the formulation and implementation of learning strategies. Given these circumstances, a highly accurate oncology system powered by artificial intelligence will be readily accessible as needed.

**Fig. 5:**
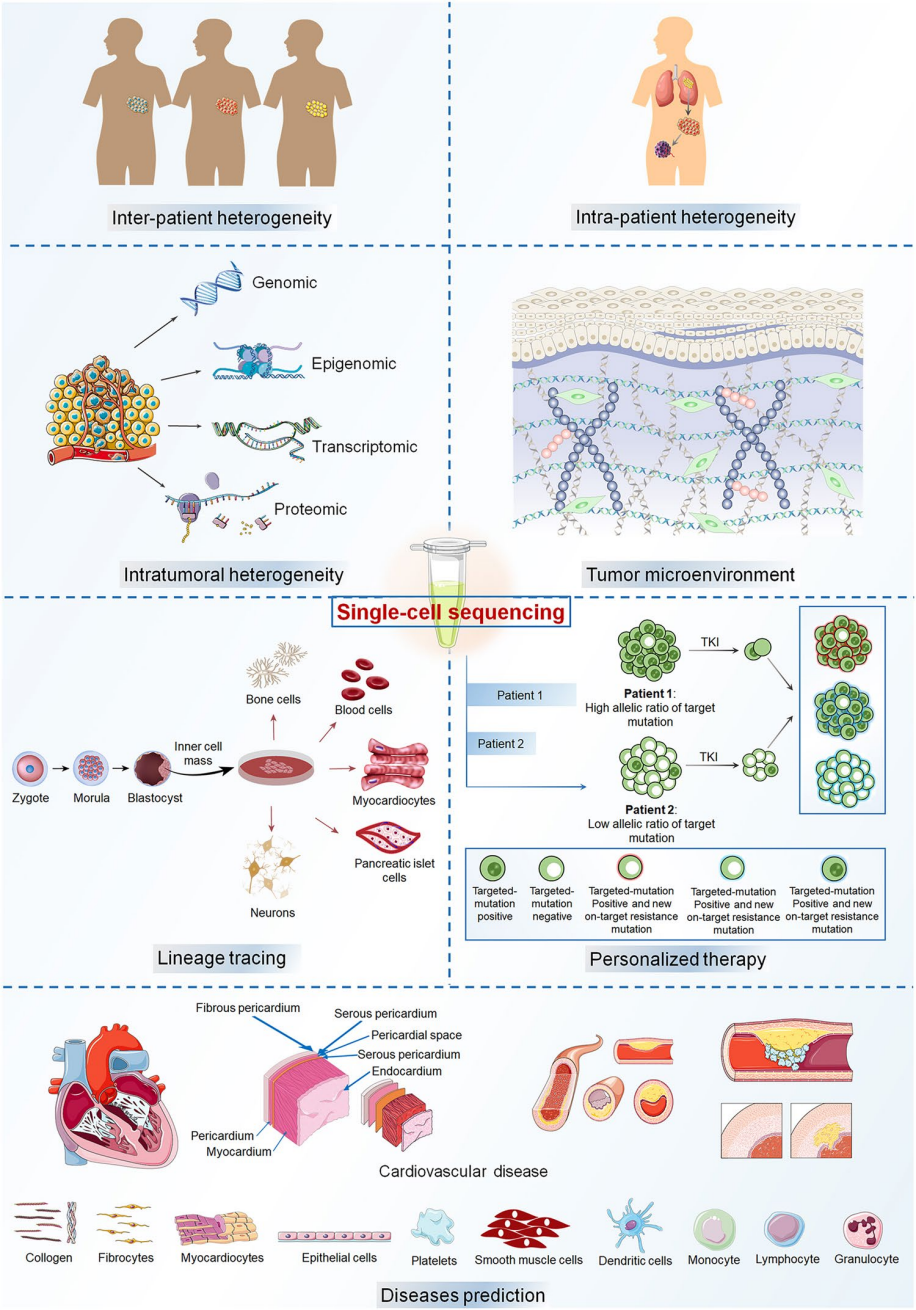
Illustrations of single-cell RNA sequencing applications in different fields. (1) tumor heterogeneity, (2) tumor microenvironment, (3) lineage tracing, (4) personalized therapy, and (5) disease prediction

In conclusion, the development of scRNA-seq and its continuous methodological improvement have led to important medical discoveries. scRNA-seq technology has been widely used in many aspects, including early mammalian embryology, tissue and organ development, the immune system, cancer, microorganism, infectious disease, and stem cell research. High-throughput scRNA-seq techniques not only reveal cellular heterogeneity during disease progression and in the immune microenvironment but also contribute to further studies on disease turnover, thus guiding early clinical diagnosis, targeted treatment, curative monitoring, and prognostic evaluation of diseases. It is expected that scRNA-seq will soon achieve 100% coverage and accuracy. Due to the rapid development of multi-omics scRNA-seq technology, single-cell genome, transcriptome, epigenome, and proteome analyses are expected to be performed simultaneously. In addition, spatially resolved transcription techniques can determine the spatial organization of cells in tissues, revolutionizing the study of tissue function and disease pathogenesis [3]. In fact, spatially resolved transcriptomic research has become a new field. Moreover, the integration of scRNA-seq and AI will become a trend in NGS. scRNA-seq will become an indispensable technology to help in more easily treating diseases and exploring the life sciences (Fig. 5).
